# Evolutionary origins and modern rise of allergic diseases: an integrative framework

**DOI:** 10.3389/fimmu.2026.1971408

**Published:** 2026-10-26

**Authors:** Ido Brami, Yuval Tal, Oded Shamriz

**Affiliations:** 1 Allergy and Clinical Immunology Unit, Department of Medicine, Hadassah Medical Organization, Faculty of Medicine, Hebrew University of Jerusalem, Jerusalem, Israel; 2 The Lautenberg Center for Immunology and Cancer Research, Institute of Medical Research Israel-Canada (IMRIC), Faculty of Medicine, Hebrew University of Jerusalem, Jerusalem, Israel

**Keywords:** allergy incidence, damage sensing, evolutionary hypotheses, hygiene hypothesis, immune surveillance, parasite defense

## Abstract

Allergic diseases have become increasingly prevalent in recent decades, prompting significant research into their evolutionary origins and the mechanisms that drive these immune responses. A range of evolutionary models has been developed to account for both the emergence and modern rise of allergic conditions. The main explanations encompass parasite defense, reduced microbial exposure and hygiene-related factors, mechanisms of tissue damage detection, and immune surveillance acting as an early warning system. Explaining why the immune system reacts inappropriately to harmless antigens demands an evolutionary lens: one that integrates ancestral adaptive roles with present-day environmental shifts. Rather than proposing a new evolutionary theory, this narrative review synthesizes competing and complementary evolutionary explanations for allergic type 1 hypersensitivity, IgE-mediated disease within a unified immunological framework, thus emphasizing recent advances in epithelial biology, immune surveillance, and type 2 inflammation that have emerged over the last decade.

## 1 Introduction

### 1.1 Historical background

Historical records suggest that one of the earliest accounts of allergy may trace back to Pharaoh Menes, who is thought to have died from an insect sting in 2641 BCE (1). By the early 19th century, John Bostock formally described hay fever, linking seasonal nasal and ocular symptoms to pollen exposure (2). Later, in 1868, Henry Hyde Salter was the first to report the presence of eosinophilia in the sputum of a patient with an allergic condition (3). The conceptualization of allergy advanced rapidly thereafter, with Clemens von Pirquet coining the term ‘allergy’ in 1906 (4), and in 1911, Leonard Noon established allergen immunotherapy by the therapeutic use of low-dose pollen extracts to treat pollen-induced allergic rhinitis (5). In 1937, Daniel Bovet synthesized antihistamines, a breakthrough that earned him the Nobel Prize in 1957 and introduced a mainstay of clinical allergy management that persists today (6). In 1953, James F. Riley identified that mast cell granules are the primary source of histamine in the body (7). These developments established the basic therapeutic approaches for allergic diseases, though the underlying mechanisms remained elusive until 1966, when Kimishige and Teruko Ishizaka discovered immunoglobulin E (IgE) as the key serum factor responsible for allergic reactions, revolutionizing serological research in allergy (8). Considerable advances have occurred since then through meticulous laboratory and clinical research, unraveling new diagnostic and therapeutic applications in allergic diseases.

### 1.2 The main players in the pathophysiology of allergy and type 2 inflammation

Allergic disease should be considered within the broader context of a dynamic immune and tissue network rather than as the consequence of a single immune pathway. Epithelial cells, innate and adaptive immune populations, tissue-resident cells, cytokines, antibodies, and metabolic signals interact bidirectionally in response to environmental and endogenous stimuli. In this context, type 2 inflammation may participate in both the initiation and propagation of allergic pathology, but the presence of a type 2 immune signature does not by itself establish whether a particular component represents an initiating event, a disease-maintaining mechanism, or a downstream response. Similarly, biomarkers identified during allergic disease may reflect pathogenic processes as well as compensatory or secondary responses. Allergy is a result of excessive immune processes encompassing parallel activation of cytokines, chemokines and repeated immunological interactions targeted to potentially heal tissue infected by helminths, as first proposed by the observations of eosinophilic pneumonia by Willem Löffler (9). The expulsion of helminth larvae displays the defining features of a type 2 immune reaction, driven by a flood of molecular interactions that enable the coexistence of the parasite and its host (10). Type 2 immunity is characterized by cytokines such as IL-4, IL-5, IL-13, which are pivotal for allergy development with activation of type 2 innate lymphoid cells (ILC2s), Th2 cells and dendritic cells (11). Epithelial cells and ILCs act as primary immunological sentinels; while the epithelium uses pattern recognition receptors and alarmins like TSLP, IL-33, and IL-25 to signal distress, ILC2s respond to these cues to initiate sensitization and work alongside DCs to drive Th2 differentiation (12). IL-4 and IL-13, produced mainly by Th2 cells and ILC2s, activate the JAK-STAT6-GATA3 pathway and promote IgE production (10). IL-5 regulates eosinophil growth, survival and migration, and enhances mucus secretion (13). Eosinophil-Derived Neurotoxin (EDN) produced by eosinophils helps DC maturation through activation of TLR2 pathway eventually enhancing T_H_2 immune responses (14, 15). These cytokines altogether are major contributors to allergic disease pathogenesis and sustain the type 2 immune response.

Regulation of allergic reaction is mediated by a diverse group of specialized immune cells mainly by regulatory T cells (Tregs). Tregs are a regulatory CD4^+^ subset expressing the Forkhead box P3 (FoxP3) transcription factor, these cells can originate from the thymus upon self-antigens stimulation (tTregs) or in specialized peripheral tissues (pTregs) from naïve CD4^+^ T cells upon foreign antigenic stimulation (16).Other cells which take part in mitigating the type 2 immune response include: regulatory B cells (Bregs), regulatory NK (NKreg) cells, regulatory DCs (DC-regs), and regulatory ILCs, each of these subsets regulates the allergic cascade in different phases or simultaneously (10). Ultimately, the strategic involvement of these various regulatory lineages across different phases of the allergic cascade underscores the evolutionary importance of maintaining strict immunological thresholds in the face of environmental allergens.

### 1.3 The rising burden of allergy

The increasing global incidence of allergy represents a significant public health issue and targeted therapeutic challenge (17). Over the past few decades, epidemiological studies have documented a substantial rise in the prevalence of allergic conditions, especially in industrialized countries (18). Factors possibly contributing to this trend include lifestyle changes, urbanization, reduced family size, diet, decreased exposure to environmental microbes, and increased pollution (19–23).The mismatch between contemporary living environments and those in which human immune systems evolved likely plays a critical role in these patterns, leading to the increase in incidence and prevalence of allergic diseases. This mismatch occurs when ancestral alleles persist in a changed environment before genetic adaptation has occurred (24). However, the extent to which these changes explain temporal or geographic variation in allergic disease remains uncertain. Current evidence therefore supports examining these relationships as testable hypotheses rather than assuming a direct causal pathway. For example, a recent systematic review concluded that indoor exposure to damp or mould increases the risk of new-onset wheeze (moderate certainty), while exposure to cleaning agents, pesticides, and volatile organic compounds (VOCs) may elevate the risk of new-onset asthma, though the evidence regarding their overall impact on pre-existing asthma outcomes remains uncertain and of low certainty (25). Another example, boys are more likely to develop childhood asthma compared with girls, at least until the point of puberty, where asthma becomes more prevalent in young women, this may be explained by differences in airway development, resistance, lung size, hormones, or even differences in consultation practices and health-seeking behavior (26).

According to data from the US National Health Interview Survey, in 2021, nearly one in five children experienced seasonal allergies, while approximately one in ten reported eczema, and around 6% had food allergies. Boys (20.0%) were more likely to have a seasonal allergy than girls (17.7%) (27). Adults showed higher rates of seasonal allergies (about 26%), but lower prevalence of eczema compared to women and older individuals, and the prevalence of food allergy decreased with increasing age (27, 28). Food allergy (FA) is recognized as a significant global public health concern (17), with higher rates observed in developed countries and urban regions (29), but evidence for a direct causal relationship between ancestral dietary patterns and contemporary food allergy remains limited. Asthma and atopic dermatitis (AD) contribute significantly to global morbidity, with rising prevalence and incidence but declining age-standardized rates in the last three decades (18).

The long-recorded history of allergy and its rising incidence indicate that this process has some biological evolutionary rationale. Despite the increase in allergy prevalence, the precise initiators of type 2 inflammation, induced by a wide range of stimuli with seemingly unrelated characteristics, remain unclear (30). Discussed hypotheses are presented in **Figure 1**.

**Figure 1:**
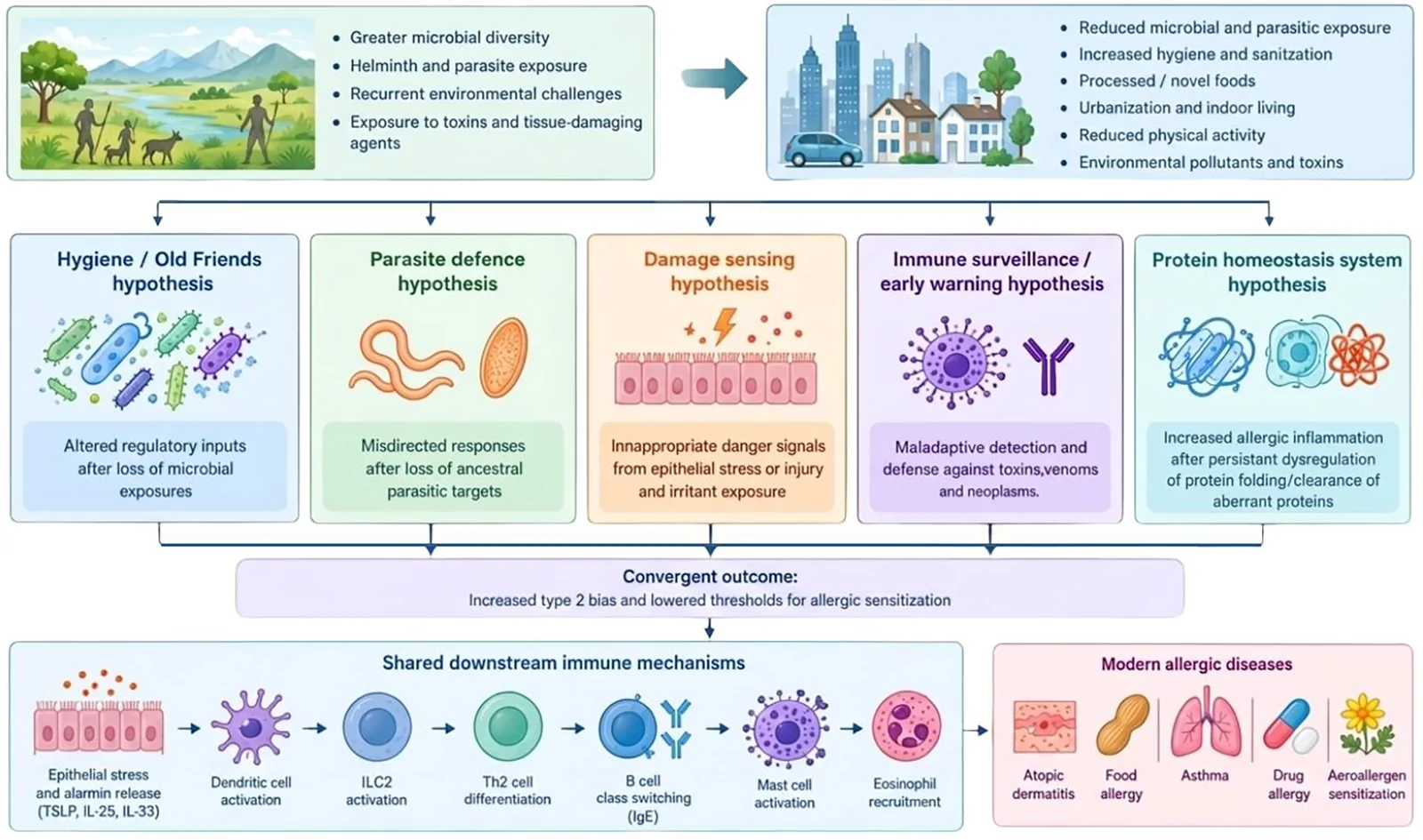
Integrative model of evolutionary hypotheses of allergy and type 2 inflammation. The five major evolutionary hypotheses, Hygiene, Parasite Defence, Damage Sensing, Immune Surveillance/Early Warning, and Protein Homeostasis, converge on a shared core of type 2 inflammation, providing a hierarchical framework that links evolutionary pressures to modern allergic disease. The model connects five mechanistic modules that translate these evolutionary pressures into contemporary clinical outcomes. The bottom panel illustrates how type 2 inflammation is sustained across allergic conditions by coordinated interactions among IgE, mast cells, eosinophils, Th2 cells, and ILC2s. DC, dendritic cell; ILC2, type 2 innate lymphoid cells; IL, Interleukin; TSLP, Thymic Stromal Lymphopoietin; DAMPs, Damage-Associated Molecular Patterns; PRR, Pattern Recognition Receptor; Th2, T helper 2 cell; IgE, Immunoglobulin E. This figure was created in https://BioRender.com. .

## 2 Possible hypotheses for the evolution of allergy

Rather than representing mutually exclusive explanations, the proposed hypotheses may reflect complementary aspects of a shared evolutionary–immunological process. Changes in environmental exposures, immune regulation, tissue integrity, and threat detection may interact to shape the activation and persistence of type 2 immune responses, potentially representing a critical link between evolutionary adaptation and modern allergic disease. Our aim is therefore to integrate these perspectives, clarify their mechanistic relationships and points of divergence, as shown in **Table 1**, and identify how their convergence may provide an explanation for the evolution of modern IgE-mediated allergic disease.

**Table 1:** Evolutionary hypotheses for allergic disease.

Hypothesis	Proposed mechanism	Strength of evidence	Key supporting evidence	Major limitations	Clinical relevance
Hygiene/Old Friends hypothesis	Reduced microbial diversity and altered early-life exposures leads to impaired immune regulation/tolerance and results in increased susceptibility to type 2 inflammation and allergy.	Moderate	Epidemiological associations with farming, sibship and microbial exposure; microbiome studies; animal models demonstrating effects of microbial colonization on immune development (31).	Human causality remains difficult to establish; effects are heterogeneous and confounded by diet, socioeconomic factors and lifestyle. The original hygiene hypothesis has evolved considerably beyond simple “cleanliness.”	Supports investigation of early-life microbial exposures, with microbiome-directed prevention and environmental interventions.
Parasite defense hypothesis	Loss of ancestral parasitic targets may leave potent type 2 effector mechanisms without their original targets, increasing responses to harmless antigens.	Moderate–strong for evolutionary plausibility; weaker for direct causation of modern allergy	Helminths induce robust type 2 responses but also powerful regulatory mechanisms; inverse associations between some parasitic exposures and allergy have been observed (32).	Helminth exposure can sometimes increase rather than decrease allergic responses; effects depend on species, intensity, timing and host context.	Provides rationale for studying helminth-derived immunoregulatory molecules and mechanisms of immune tolerance.
Damage Sensing hypothesis	Type 2 immunity may have evolved too rapidly to detect tissue injury propelled from epithelial stress/damage with DAMP/PRR signaling and alarmin release induce ILC2/DC activation and type 2 inflammation propagation.	Moderate mechanistic evidence; limited direct evolutionary evidence	Strong experimental evidence linking epithelial injury, barrier disruption and alarmins to type 2 inflammation (33).	Demonstrates how allergy can be initiated but does not by itself establish why this system evolved or why modern allergy increased.	Directly relevant to epithelial-barrier therapies, alarmin-targeted treatment and environmental exposure research.
Immune Surveillance/Early Warning hypothesis	Highly sensitive IgE/mast-cell responses provide rapid threat detection; in modern environments, this sensitivity may be misdirected toward innocuous antigens.	Moderate and rapidly expanding	Experimental toxin/venom studies, mast-cell biology, IgE-mediated surveillance and emerging allergo-oncology evidence (34).	Much of the evolutionary argument remains inferential; clinical associations between allergy, IgE and cancer are complex and not uniformly protective.	Relevant to understanding IgE biology, allergy therapeutics and emerging IgE-based cancer immunotherapy.
PHS hypothesis	Etiological or inflammation- inducing substances may be small molecules derived from outside of the host such as chemicals and biochemicals reacting with corresponding immune components	Emerging/preliminary	Clinically, most patients with allergic diseases are highly sensitive to Cs, and Cs may work against non-specific immune clones in allergies (35).	Etiological substances and corresponding immune components in allergies are not clearly defined at present time. Immune systems may have evolved to control toxic substances both external and internal origin.	Could provide new targets for understanding disease persistence, severity and therapeutic intervention.

PHS, Protein-Homeostasis-System, DAMP/PRR, Damage-Associated Molecular Pattern/Pattern Recognition Receptor; ILC2, type 2 innate lymphoid cells; DC, Dendritic cells; IgE, Immunoglobulin E; Cs, corticosteroids.

### 2.1 Hygiene hypothesis

The Hygiene Hypothesis was first introduced by Strachan in 1989, after increasing family size was associated with a reduced risk of developing hay fever (36). However, earlier work by Peter John Preston and John Turton had already linked changes in the human-associated biota to allergic disease, including the hygiene-associated loss of complex eukaryotic symbionts, particularly helminths (37, 38). This theory is supported by evidence from several studies, which show lower allergy rates in children raised on farms or with more siblings and especially older siblings, as well as the protective effects of early-life exposure to microbial diversity (39–44). Westernized lifestyles have shifted people indoors, increasing exposure to indoor allergens, microbes, and chemicals, which in turn affects the risk and development of allergic diseases (21, 45). Furthermore, improved sanitation, and reduced exposure to a diversity of microbes during childhood has been associated with altered immune development and may influence Th2-skewed responses (46).

While lifestyle changes strongly correlate with altered incidence of allergic and autoimmune diseases, causality remains unproven amid confounders like diet, healthcare quality, and regional habits (47).

This theory is supported by the alteration of the immune system seen in germ-free murine model (48), and limited clinical interventions provide key causal evidence.

Modern diet may have an impact on inflammation, as Ultra-processed foods (UPFs) now constitute a prominent and widely consumed component of contemporary diets (49). A recent study using data from 11,765 participants in the National Health and Nutrition Examination Survey (NHANES) found that higher intake of UPFs was dose-dependently associated with increased odds of self-reported food allergy, asthma, and wheeze, even after adjustment for dietary fiber, antioxidant intake, and BMI, thereby supporting a link between modern diet and allergic disease (50).

Another immunological explanation may originate during the prenatal period, when the fetus, which contains paternally derived cells and antigens, can be viewed as a semi-allogeneic graft within the mother. This state may promote immune tolerance through enhanced regulation and suppression of Th1 responses, accompanied by a relative upregulation of Th2 immunity (31).Thus, maternal and infant microbial diversity is vital for healthy immune development (51), as metabolites like short-chain fatty acids and other metabolites produced by maternal gut microbes influence fetal immune cell populations (52).One example is the research conducted within the CHILD birth cohort (Canadian Healthy Infant Longitudinal Development study) utilized shotgun metagenomic sequencing and metabolomic profiling of 1115 infant stool samples at 3 months and 1 year to demonstrate that delayed gut microbiota maturation characterized by dysbiosis, reduced protective species, mucous degradation, oxidative stress, and elevated trace amines as a shared feature was associated with subsequent diagnoses of atopic dermatitis, asthma, food allergy, and allergic rhinitis by the age of 5, supporting the hypothesis that altered microbiota development may contribute to allergic disease risk (53).

A recent meta-analysis found that maternal antibiotic use during pregnancy appears to be associated with an increased risk of wheeze, asthma, and other diseases along the atopic march in offspring, although substantial heterogeneity and overall low-quality evidence underscore the need for more rigorous studies in this area (54).Another meta-analysis of 113 studies demonstrated that offspring delivered via cesarean section face a significantly elevated risk for a broad spectrum of allergic conditions, including asthma, food allergies, allergic rhinitis, atopic dermatitis, and allergic sensitization, compared to those delivered vaginally (55).

To fully acknowledge the hygiene hypothesis, studies must cover longitudinal monitoring from prenatal stages through later development, as early shifts in microbiota composition can drive immune imbalance, predisposing children to atopic conditions that may continue into adulthood.

### 2.2 Parasite defense hypothesis

The Parasite Defence Hypothesis posits that allergic responses originally evolved as a protective mechanism against macro-parasites, such as helminths, and environmental toxins (56),as the prevalence of asthma, eczema, and urticaria was greater in communities less exposed to helminth infestations and other untreated viral and bacterial infections (57). The decline in parasite prevalence in industrialized societies (31), may leave these powerful immune mechanisms without their historical targets, resulting in a fitness “cost” of uncontrolled IgE-mast cell axis that now responds to otherwise innocuous antigens, manifesting as allergic disease. Helminth infections induce robust Type 2 (Th2) immune responses, characterized by IgE production and mast cell recruitment (58), but they also evoke regulatory pathways that dampen overactive inflammation (46). Helminths secrete anti-inflammatory molecules that induce regulatory T cells (Tregs), regulatory B cells, type 2 macrophages, and tolerogenic dendritic cells, resulting in immune suppression (32). For example, *Ascaris* spp. and *Strongyloides* spp. infections predominantly elicit type 2 immune responses, by inducing secretion of IL-4, IL-5, IL-9, and IL-13 and lower levels of type 1 cytokines like IFN-γ and TNF-α, whereas tissue-dwelling nematodes induce a more mixed profile characterized by prominent Treg activity with the production of regulatory cytokines, such as IL-10 and TGF-β, alongside both type 2 and type 1 responses (59). Given helminths’ potent immunoregulatory capabilities, there is active research into harnessing worm-derived molecules and even controlled helminth exposure for the reprogramming of allergies, autoimmune diseases, and, as recent evidence suggests, certain cancers (60).

### 2.3 Damage sensing hypothesis

The Damage Sensing Hypothesis proposes that allergic inflammation is triggered not solely by foreign antigens, but through immune detection of tissue stress or damage (33). Allergic mechanisms may provide rapid detection and alerting functions in response to potential environmental dangers, such as venomous bites, stings, or harmful airborne substances, however, in modern sterile environments, this adaptive mechanism is more likely to misfire. Recent studies have accomplished significant advances in understanding different mechanisms such as physical epithelial barrier breach, cell stress pathogen recognition receptor (PRR) systems and damage-associated molecular patterns (DAMPs) as inflammation drivers which orchestrate allergic responses. Disruption of the epithelial barrier activates neighboring immune cells, including macrophages, ILCs, and mast cells, initiating a type 2 inflammatory response and promoting the subsequent development of allergy (61).

In asthma, the structural and immunological integrity of respiratory epithelial cells, comprising ciliated, goblet, club, and basal cells connected by tight and adherence junctions and coated with mucus rich in antimicrobial agents, peptides, and antibodies, plays a crucial role in protecting against pathogens and allergens and maintaining airway homeostasis (62, 63).In individuals with asthma, prolonged exposure to environmental stimuli leads to epithelial barrier dysfunction characterized by an increase in basal and goblet cells and a reduction in terminally differentiated ciliated cells (64).

The emergence of air-pollution–associated allergic disease as a contemporary and rapidly evolving threat may reflect a shift from sustained exposure toward recurrent, intermittent exposure patterns. This change may be driven by modern lifestyles characterized by increased time spent indoors, widespread use of air conditioning, sedentary behavior, and reduced mobility and travel, resulting in repeated transitions between different environmental exposures.

In food allergy, impaired intestinal epithelial junctions cause barrier and osmotic dysfunction, increasing permeability that permits allergen penetration and alarmin release (TSLP, IL-25, IL-33), which activates cutaneous dendritic cells and type 2 ILCs, drives IL-4 and IL-13 production, and shifts the immune response from tolerance to hypersensitivity (65). The emergence of food allergy may partly reflect an evolutionary mismatch between ancestral dietary exposures and the increasingly diverse food environment created by globalization. Populations adapted to relatively stable, region-specific diets are now exposed to novel foods and food components that were historically absent or uncommon in their ancestral environments, potentially challenging established mechanisms of oral tolerance and contributing to aberrant immune sensitization.

To test this novel hypothesis, longitudinal clinical studies should quantify tissue barrier integrity, epithelial stress signals and alarmin signatures, and ILC2 activation transcriptomes to predict allergy onset more effectively than antigen exposure alone.

### 2.4 Immune surveillance and early warning hypothesis

Allergic immunity is increasingly recognized as an evolutionarily conserved early warning and immune-surveillance mechanism, as first proposed by Profet in 1991 (66), and supported by experimental evidence demonstrating innate and adaptive resistance to venoms and toxins instead of pure pathological processes (67),functioning as a localized alarm system that detects epithelial damage or chemical irritants (68). Mast cells and other innate immune cells detect and respond to signals of damage or disruption, amplifying their response to environmental triggers along with adaptive T and B-cell immunity, especially IgE antibody production (58).

IgE-mediated responses and the activation of mast cells are highly effective at expelling parasites and neutralizing toxins, providing a strong selective advantage in ancestral environments. First, IgE-sensitized mast cells act rapidly to minute quantities of an allergic antigen, conferring a form of anticipatory defense against repeated venom or toxin exposure. Second, IgE-dependent mechanisms of mast cell activation functions as a form of antibody-dependent cell-mediated cytotoxicity (ADCC) akin to NK cell activity; this process induces phagocytosis and supports a broad immune surveillance role beyond classic allergy and is now known to play a pivotal role in autoimmunity and chronic inflammatory diseases (69). For example, more than 140 and 200 autoantigens recognized by IgE have been detected in patients with atopic dermatitis and chronic spontaneous urticaria (70).

Enhanced immune alertness have offered survival benefits, if the offending antigen is constantly present, however in modern days where therapeutic intervention is immediate and the environment is more sterile, it can be maladaptive when triggered by harmless modern allergens. Immune surveillance and early warning in allergy are increasingly recognized as dynamic contributors to cancer biology, shaping the emerging field of allergo-oncology. Epidemiological and mechanistic studies suggest that certain allergic diseases and heightened IgE responses can potentially decrease cancer risk through enhanced immunosurveillance and immune hyperresponsiveness (34). The immune phenomena underlying allergy may both protect against and promote tumorigenesis (71). A recent large meta-analysis showed significant negative correlations between different allergies and cancers such as colorectal cancer, lymphoma, pancreatic cancer, leukemia, and brain cancers, while increase risk of lymphoma in atopic dermatitis patients (72). Mast cells and eosinophils, abundant in allergic inflammation, also contribute to anti-tumor immunity by releasing cytotoxic mediators and recruiting additional immune cells. High serum IgE and activation of allergy-specific effector cells may participate in tumor immunosurveillance, identifying and eliminating aberrant cells at an early stage (34, 73).

Experimental models show that IgE ablation impairs antitumor immunity (71). Adults with IgE deficiency were shown to suffer from autoimmune diseases (74) and higher incidence of cancer (75). A recently published population-based study in children with up to 5 years of follow-up revealed that IgE deficiency resulted in increased risk for solid tumors, autoimmune disorders, and inborn errors of immunity (76). IgE-based immunotherapies represent a novel therapeutic avenue in oncology, leveraging the heightened effector functions of IgE to activate anti-tumor immune responses, and were proved efficacious in suppressing tumor growth in both rodents and early-phase clinical trials (77, 78).

### 2.5 Protein-homeostasis-system hypothesis

The protein homeostasis system (PHS) hypothesis, recently proposed as a unifying framework for allergic diseases (35), suggests that there are etiological substances in each allergy and corresponding immune components, such as mast cell, IgE, and eosinophils. Inflammation caused by these immune reactions may induce type 2 inflammation and target cell injury including epithelial cell damage. This emerging hypothesis proposes that allergic disease may partly reflect disturbances in protein homeostasis (proteostasis), the cellular processes that maintain the synthesis, folding, modification, and clearance of proteins. On the other hand, studies on relationship between proteostasis and allergies suggested that impaired proteostasis can generate cellular stress and inflammatory signaling (79), including activation of the unfolded-protein response in allergic airway disease (80). Human studies have further linked endoplasmic-reticulum stress with local IgE production in allergic rhinitis (81), while *in vivo* experimental data indicate that leptin induces expression of the unfolded protein response factor XBP1s in pro-allergic Th2 cells as mice fed a high-fat diet displayed increased serum leptin concentrations, comparable to those seen in obese human populations, and showed worsened asthmatic symptoms concomitant with upregulated XBP1s expression in splenic CD4^+^ T cells (82). Together, these findings raise the possibility that impaired handling of proteins and bioactive molecules may not simply accompany allergic inflammation but could contribute to its persistence and progression. Importantly, whether altered proteostasis represents an initiating mechanism or a consequence of established allergic inflammation remains uncertain and requires further prospective and mechanistic investigation.

## 3 Limitations

Our review has several limitations. We employed a narrative rather than a systematic review methodology, acknowledging the potential for selection and publication bias and the heterogeneity of evidence across evolutionary hypotheses. Much of the evolutionary argument relies on mechanistic, observational, and animal-study evidence rather than direct demonstrations of evolutionary causality, as causal relationships between environmental changes and the development of allergic disease cannot be directly established from the available evidence. Some hypotheses, such as the immune-surveillance and damage-sensing hypotheses, overlap mechanistically and may not be readily distinguishable experimentally. Moreover, evolutionary explanations are necessarily partly inferential and should therefore be regarded as conceptual frameworks and testable hypotheses rather than definitive explanations of allergy. Future prospective and experimental studies will be required to evaluate these hypotheses and determine their relative contribution to allergic disease.

## 4 Conclusion

Allergies arise from a complex interplay between evolutionary adaptations and modern environmental factors, further amplifying current allergy prevalence. Understanding these hypotheses’ interplay provides crucial insight into allergy prevention and management, guiding future epidemiological strategies and research on both the origins of allergy and as a modern epidemic.
